# Design Process and Utilization of a Novel Clinical Decision Support System for Neuropathic Pain in Primary Care: Mixed Methods Observational Study

**DOI:** 10.2196/14141

**Published:** 2019-09-30

**Authors:** Dale Guenter, Mohamed Abouzahra, Inge Schabort, Arun Radhakrishnan, Kalpana Nair, Sherrie Orr, Jessica Langevin, Paul Taenzer, Dwight E Moulin

**Affiliations:** 1 Department of Family Medicine McMaster University Hamilton, ON Canada; 2 College of Business California State University Seaside, CA United States; 3 Department of Family and Community Medicine University of Toronto Toronto, ON Canada; 4 School of Nursing McMaster University Hamilton, ON Canada; 5 Department of Physical Medicine and Rehabilitation Queen's University Kingston, ON Canada; 6 Department of Clinical Neurological Sciences Western University London, ON Canada; 7 Department of Oncology Western University London, ON Canada

**Keywords:** medical records systems, computerized, quality of health care, pain management, medical informatics

## Abstract

**Background:**

Computerized clinical decision support systems (CDSSs) have emerged as an approach to improve compliance of clinicians with clinical practice guidelines (CPGs). Research utilizing CDSS has primarily been conducted in clinical contexts with clear diagnostic criteria such as diabetes and cardiovascular diseases. In contrast, research on CDSS for pain management and more specifically neuropathic pain has been limited. A CDSS for neuropathic pain has the potential to enhance patient care as the challenge of diagnosing and treating neuropathic pain often leads to tension in clinician-patient relationships.

**Objective:**

The aim of this study was to design and evaluate a CDSS aimed at improving the adherence of interprofessional primary care clinicians to CPG for managing neuropathic pain.

**Methods:**

Recommendations from the Canadian CPGs informed the decision pathways. The development of the CDSS format and function involved participation of multiple stakeholders and end users in needs assessment and usability testing. Clinicians, including family medicine physicians, residents, and nurse practitioners, in three academic teaching clinics were trained in the use of the CDSS. Evaluation over one year included the measurement of utilization of the CDSS; change in reported awareness, agreement, and adoption of CPG recommendations; and change in the observed adherence to CPG recommendations.

**Results:**

The usability testing of the CDSS was highly successful in the prototype environment. Deployment in the clinical setting was partially complete by the time of the study, with some limitations in the planned functionality. The study population had a high level of awareness, agreement, and adoption of guideline recommendations before implementation of CDSS. Nevertheless, there was a small and statistically significant improvement in the mean awareness and adoption scores over the year of observation (*P*=.01 for mean awareness scores at 6 and 12 months compared with baseline, for mean adoption scores at 6 months compared with baseline, and for mean adoption scores at 12 months). Documenting significant findings related to diagnosis of neuropathic pain increased significantly. Clinicians accessed CPG information more frequently than they utilized data entry functions. Nurse practitioners and first year family medicine trainees had higher utilization than physicians.

**Conclusions:**

We observed a small increase in the adherence to CPG recommendations for managing neuropathic pain. Clinicians utilized the CDSS more as a source of knowledge and as a training tool than as an ongoing dynamic decision support.

## Introduction

### Background

Computerized clinical decision support systems (CDSSs) can be defined as the information and communication systems that provide clinicians or patients with timely, accurate, and appropriate knowledge to enhance patient care [1]. They have emerged as an attractive approach to improving compliance of clinicians with clinical practice guidelines (CPGs). Their potential to improve knowledge translation of the CPGs is being considered for an increasing variety of clinical contexts. However, their effectiveness in achieving this continues to be controversial.

Evidence describing the effectiveness of CDSSs in improving knowledge translation comes from a number of systematic reviews, including studies carried out in a variety of contexts [2-5]. The majority of studies reviewed were conducted in outpatient and academic settings.

The effectiveness of CDSSs in these settings is supported through improvements in process adherence, including medication ordering, vaccinations, test ordering, and diagnosis and disease management [3-5]. However, not all clinical processes have shown improvements after implementing a CDSS [3,4]. Of note is that most of the research has been related to diabetes and other cardiovascular risks, areas that have clear diagnostic criteria and well-supported management strategies and disease monitoring processes.

Pain management, on the other hand, has less clarity for many diagnostic criteria and less consensus on effective treatment and monitoring. Thus, the knowledge base is more difficult to translate. Little research has been conducted on the effect of CDSS on pain management generally, and neuropathic pain more specifically. The available literature, including 1 systematic review, is primarily focused on cancer pain management [6-11]. To a lesser extent, CDSS pain research has been conducted around chronic noncancer pain, including headaches, as well as low back pain (LBP) and neuropathic pain [6,12]. A systematic review emphasized the need for further research in this area as all studies included were nonexperimental [6]. Although a more recent experimental study examined the impact of a CDSS on the outcomes of patients with chronic pain in primary care, results demonstrated that these patients were still undertreated or inadequately treated [12].

Factors that limit and facilitate the effectiveness of CDSS have been reported. Most studies are able to demonstrate improvements in clinical process, but very little data provide information about improved patient outcomes [6]. Many patient-specific outcomes for chronic pain have not been explored, including health care utilization, health care costs, pain relief, pain medication usage, communication with providers, functional status, and quality of life. Barriers to effectiveness include inadequate training, poor usability or integration into practice workflow, and nonacceptance by practitioners of computerized recommendations [13-15]. Success has been more common in systems that prompt users to use the tools, those that have been developed by trial authors rather than externally and those that provide recommendations and not only assessments [2].

The Veterans Affairs (VA) in the United States developed a CDSS for neuropathic pain in their national electronic medical record (EMR) that involved significant clinician engagement in prototype development to improve use [16]. Although the significant clinician engagement resulted in improvements in the focus, scope, content, and presentation of the CDSS, the effects on patient outcomes were not evaluated [16]. In addition, the VA neuropathic pain CDSS is no longer active as frequent updates were required to maintain current clinical knowledge, which was not possible once the research project ended [17].

Neuropathic pain is a unique subset of pain conditions that often becomes chronic, decreasing function and quality of life [18,19]. It can be difficult to diagnose even though it has specific diagnostic criteria. Treatment is also unique in that pain responds best to medications that are used mainly to treat seizures or to treat depression. It is defined by the International Association for the Study of Pain as “pain caused by a lesion or a disease of the somatosensory system” [20]. The estimated prevalence of neuropathic pain in the general population is 2% to 3%; however, there are estimates that 7% to 8% of the population experience pain with neuropathic components [20].

Overall, the challenge of diagnosing and treating patients with neuropathic pain often leads to tension in clinician-patient relationships, frequent and prolonged visits with high emotional intensity, poor patient compliance, poor clinical outcomes, and sometimes refusal of access to care for those who identify as having chronic pain. It is clear that support is needed to facilitate optimal care for patients with chronic pain in primary care [21].

### Objectives

Our aim was to improve the adherence to CPG recommendations in primary care for the diagnosis and treatment of neuropathic pain through CDSS. The CDSS development has been reported previously [22]. Our hypothesis was that improved management would result from a tool with high usability combined with recommendations that were acceptable to clinicians. We sought to answer the following research questions: (1) How does self-reported awareness of, agreement with, and adoption of key CPG recommendations for neuropathic pain change among clinicians during the year following the introduction of the CDSS? and (2) How does the observed adherence to key CPG recommendations for neuropathic pain change from 1 year preceding to 1 year following the introduction of the CDSS?

## Methods

### Study Setting

The CDSS and the evaluation study were both developed through engagement of an advisory board made up of clinician researchers, software developers, end users, CPG developers, and information systems experts. The project was based at the Department of Family Medicine at McMaster University in Hamilton, Canada. The CDSS was designed for the Open Source Clinical Application Resource (OSCAR) EMR [23]. At the time this project was launched, an overhaul of the user interface for OSCAR was in process, presenting the opportunity for the function of the CDSS within the EMR to be optimized.

Overall, 3 academic family medicine clinics, all involved in delivering McMaster’s family medicine residency training program, were the sites where development, testing, implementation, and evaluation took place. Together, these clinics serve 40,000 patients, with 169 clinicians, including family physicians, family medicine residents, and nurse practitioners.

The study was approved by the Hamilton Integrated Research Ethics Board, reference number 13-136.

### Stakeholder Consultation and Usability Testing for Clinical Decision Support System Requirements

We began with a broad consultation with the varied stakeholders on the advisory board, as described above. This was followed with focus group discussions with interprofessional end users to determine the requirements for the CDSS content, appearance, function, and workflow. Practice recommendations were drawn from the Canadian guidelines for management of neuropathic pain [24]. The consultation process informed the development approach outlined below. A prototype was developed and followed with iterative cycles of usability testing and modification. This process has been reported previously [22].

### Clinical Decision Support System Deployment

The final version of the CDSS was created and integrated into the OSCAR EMR code. Its functionality was designed to be optimal with a new user interface that was scheduled to be in use by the time of the study. However, this did not occur and the CDSS was deployed within the older user interface environment. Although all functions were available on the older user interface, gaining access to and the appearance of the CDSS were not as clear or easy to follow as the prototype design.

### Evaluation of Clinician Awareness, Agreement, and Adoption

#### Design and Participants

We conducted a quasi-experimental study comparing clinicians at the preintervention phase (baseline) with 6 months and 12 months after the introduction of the CDSS. A questionnaire was used to assess the degree to which a clinician was aware of, agreed with, and felt they had adopted key CPG recommendations for managing neuropathic pain.

Our recruitment goal was 120 clinicians, with 50 family physicians, 50 family medicine residents (postgraduate physicians in training), and 20 nurse practitioners (advanced practice nurses). Participants were recruited to the study through presentations about the CDSS at clinician rounds and other prescheduled educational events.

#### Training of Participants

Participants were asked to attend 1 training session of 1-hour duration, use the CDSS during clinical encounters, and complete questionnaires about awareness, agreement, and adoption of guideline recommendations. They were also invited to a focus group discussion about the experience of using the CDSS. Training videos about the use of the CDSS were created and posted on the Web to be widely available. Each clinic recruited a study champion on site to promote the CDSS and address any issues about its use. A total of 12 training sessions were completed over the duration of the project.

All clinicians (both participants and nonparticipants in the study) were welcome to attend the training sessions, to use the CDSS, and to access the clinic champions. The utilization of the CDSS was also measured for all clinicians (see *Evaluation of Utilization* below). We defined *study participants* as the subset of clinicians who completed questionnaires about neuropathic pain recommendations and CDSS use.

#### Development of Questionnaire for Awareness, Agreement, and Adoption

The questionnaire measuring awareness, agreement and adoption was developed based on Pathman awareness-to-adherence model [25]. This model proposes that for clinicians to adhere to a guideline recommendation, they must first be aware of it, then agree with it, and finally adopt it into their routine practice when appropriate.

Awareness of a guideline recommendation is a measure of how much the clinician has been exposed to, and recognizes, the recommendation. Agreement is a measure of whether the clinician thinks this recommendation has value or is correct. Adoption is a measure of whether the clinician intends to use the recommended practice. Adherence is the observed, objective measure of the use of a recommended practice. The questionnaire was pretested and revised for face and content validity. Through our consultation process, the following 2 key practice guideline recommendations for neuropathic pain were chosen: (1) making a diagnosis of neuropathic pain and (2) prescribing first-line medications specific to neuropathic pain.

#### Analysis

To evaluate change in awareness, agreement, and adoption over time, we used the dependent *t* test to compare the means of these 3 constructs at time 0 and at 6 and 12 months following CDSS introduction.

### Evaluation of Clinician Adherence

Adherence is defined as objective, observed practice of the key guideline recommendations. This was measured through a visual review of the EMR records. As neuropathic pain is a relatively rare condition in primary care, and our goal was to audit a large number of clinical encounters that involved pain management, we elected to select records based on a query for clinical encounters involving pain, rather than based on the clinicians who were providing care. As the introduction of the CDSS could have an impact on all clinicians, not only those who were enrolled in the study, all patients in the clinic and their encounters with all clinicians were deemed eligible to be sampled.

We selected a sample of 100 patients for chart review before CDSS introduction and an independent sample of another 100 patients for chart review at 12 months following CDSS introduction. We used the following selection procedure. All patients in the clinic aged over 17 years with a clinic visit in the past 12 months were deemed eligible. Queries were developed to identify patients with new acute neuropathic pain or an acute exacerbation of neuropathic pain. From the list generated from the EMR, 100 patients were randomly selected for review. This same procedure was conducted at 12 months following CDSS introduction, with the removal of any patients who had been assessed already in the first selection and appearing again in the second selection.

The review of records was conducted by an independent medical doctor who was not an investigator. The reviewer used a data template to extract pertinent measures. For the first 10 patients, records were independently reviewed by one of the investigators (DG) to determine that interrater reliability reached a kappa of 0.95. All clinical encounters experienced by any clinician were reviewed.

### Evaluation of Utilization

We defined CDSS utilization as the entering and saving of data in any of the fields of any of the forms of the CDSS. Opening the form, viewing it, or entering data that were not saved was not captured in our utilization query. A query of the data saved in the CDSS was run at the 6- and 12-month time points. Provider names were recoded with study identifiers, as well as provider type. Descriptive statistics were used to analyze the patterns of utilization.

### Evaluation of User Experience of Clinical Decision Support System

A total of 5 focus groups were conducted with 2 at each of the 2 clinics and 1 at the third. All clinicians who had enrolled in the study were eligible to participate in a focus group, with the purpose of assessing satisfaction with, and overall experience of, using the CDSS. Clinicians were welcome to participate in the focus group discussion if they felt familiar enough with the CDSS to comment on their own experience. There were at least 2 research team members attending each group, and all were led by an experienced facilitator (KN). Group discussions were digitally recorded and transcribed verbatim. Overall, 2 team members (MA and KN) completed the coding and analysis of the data.

## Results

### Outcome of the Clinical Decision Support System Development Process

#### Guiding Principles for the Clinical Decision Support System Development

The consultation process resulted in the following overarching principles to guide the CDSS development:

- The pain experience comprises a variety of symptoms and issues and requires strategies and providers from various disciplines. The CDSS should include tools that address the scope and depth of the pain experience.
- The CDSS should allow assessment, monitoring, and graphing of trends for symptoms over time.
- The CDSS should decrease the burden of finding relevant historical patient data in the EMR, such as previous and current medications, diagnostic tests and consultations, and trends in symptoms over time.
- The CDSS should offer the opportunity to access as much or as little decision support as the clinician prefers.
- Clinical parameters collected in the management of other chronic conditions in primary care that may also be relevant to pain management (such as vitamin B12 level in neuropathic pain) should be imported for reference during the pain assessment. Flow sheets for multiple chronic conditions should be visible at the same time.
- The CDSS should support patients in their own self-management plans.

#### Content

Various components of the CDSS may be viewed online [26]. Separate forms or *modules* were created. The *encounter guide* offers clinicians an approach for assessment, diagnosis, and treatment of neuropathic pain. An encounter guide was also created for LBP and for opioid management as these were deemed common and often accompanied neuropathic pain. Each of these offered practice recommendations from the relevant guideline, fields to input clinical data, and options to read or to view a brief video of the supporting evidence for the recommendation. In addition, validated questionnaires were coded as tools for monitoring more general parameters about chronic pain, including pain/function levels measured by Brief Pain Inventory, sleep measured by Pain and Sleep Questionnaire three-item index, the four-item Patient Health Questionnaire for depression and anxiety, and the Primary Care PTSD Screen for trauma. Finally, a questionnaire was developed to assess goal and planning.

#### Function

Clinical parameters that may have been collected as part of the clinical care unrelated to the pain assessment encounter were imported automatically into the neuropathic pain encounter form for ease of viewing (laboratory values, demographics, vital signs, and medication lists). Values entered that required calculation to become summary values had formulas coded (scores on questionnaires, conversion to milligram equivalents of alternate opioid medication, and renal toxicity levels). Values entered or calculated that were deemed important to follow over time then populated a *health tracker* flow sheet summary, which also graphed trends. All modules could be printed for distribution, attached to referral or insurance letters, sent to personal health record, and used on mobile devices.

#### Self-Management Support

Each module had embedded links to materials that could support self-management, including videos, documents, and websites. These could be accessed during the visit or by the patient at another time. In addition, a 2-minute *in the moment* video was created to provide the main teaching points to both clinicians and patients about the key recommendations. For neuropathic pain, this included a video about the importance of making a diagnosis of neuropathic pain and a video about the unique medications used for neuropathic pain.

### Evaluation of Clinician Awareness, Agreement, and Adoption

Participants are described in Table 1. There was a population of 169 available clinicians among the 3 study clinics at baseline. Those who agreed to participate in the study by completing questionnaires included 34 family physicians, 75 residents (first year, n=64; second year, n=11), and 9 nurse practitioners, for a total of 118 of 169 clinicians, or 69.8% of available clinicians at baseline for all 3 sites.

Of the 118 clinicians who consented to participate in the study, 100 (84.7%) completed the baseline and 66 (55.9%) completed the 6-month questionnaires. There were fewer eligible participants for the 12-month time point owing to 1 clinic being delayed in starting and the second-year residents graduating before study completion. This allowed only 2 time points to be collected for those participants. Thus, there were 86 eligible participants and 35 (40%) completed questionnaires at 12 months.

Clinician awareness, agreement, and adoption of guideline recommendations was high at baseline in this study population, with mean scores in the top quartile for all parameters. Scores at 6 and 12 months when compared with baseline were, however, significantly higher for both awareness and adoption.

**Table 1:** Clinician research participants and awareness, agreement, and adoption scores over time.

Enrolled participants		0 months (N=118)	6 months (N=118)	12 months^a^ (N=86)
**Completed awareness, agreement, and adoption questionnaires, n (%)**				
	Physician	32 (27.1)	25 (21.2)	18 (20)
	Residents	59 (50.0)	34 (28.8)	11 (12)
	Nurse practitioner	9 (7.6)	7 (5.9)	6 (7)
	Total	100 (84.7)	66 (55.9)	35 (40)
**Scores for 2 neuropathic pain recommendations (0 months is comparator)**				
	Awareness^b^, mean (SD)	4.1 (0.55)	4.4 (0.54)	4.5 (0.56)
	*P* value	—^c^	.01	.01
	Agreement^d^, mean (SD)	4.4 (0.38)	4.5 (0.47)	4.5 (0.42)
	*P* value	—	.91	.91
	Adoption^e^, mean (SD)	4.2 (0.70)	4.7 (0.73)	4.6 (0.74)
	*P* value	—	<.01	.01

^a^Total enrolled at 12 months is lower because of attrition of 1 clinic and graduating residents.

^b^Familiarity with guideline recommendation on a 5-point scale.

^c^Not applicable.

^d^Agreement with guideline recommendation on a 6-point scale.

^e^Frequency of use of guideline recommendation on a 5-point scale.

### Clinician Adherence

Table 2 describes the characteristics of the patient sample populations selected to audit the adherence of clinicians to guideline recommendations before and after the introduction of the CDSS. Characteristics of pre- and postsamples were similar. The majority of the sample was female.

Table 2 also reports the degree of adherence to history, examination, and treatment recommendations for neuropathic pain. As this is a primary care population, many visits were not related to a complaint of pain, but for about half of all the visits, pain was a concern. Among the visits where pain was a concern, a significantly higher number of visits in the post-CDSS audit (50.9% [171/336] post vs 39.0% [156/400] pre; *P*=.001) described features of neuropathic pain as being either present or absent. Similarly, there was a significantly higher number of visits for which sensation testing was carried out and recorded during the post-CDSS period (35.1% post vs 12.3% pre; *P*<.001).

**Table 2:** Neuropathic pain management pre-clinical decision support system (CDSS) and post-CDSS, from chart audit.

Chart audit results		Pre-CDSS sample	Post-CDSS sample
**Demographics**			
	Total patients, N	100	100
	Sex (male), n (%)	37 (37.0)	43 (43.0)
	Age (years), mean	55	59
	Total number of visits in 1 year (all types), n	824	664
**Visits for pain management**			
	Number of visits dealing with pain, N	400	336
	Pain visits with neuropathic features asked on history, n (%)^a^	156 (39.0)	171 (50.9)
	Pain visits with neuropathic features examined physically, n (%)^b^	49 (12.3)	118 (35.1)
	Pain visits with first-line medication continued from previous, n (%)^c^	157 (39.3)	144 (42.9)
	Pain visits with first-line medication initiated, n (%)^c^	47 (11.8)	48 (14.3)
	Pain visits with second-line medication continued from previous, n (%)^d^	139 (34.8)	107 (31.8)
	Pain visits with second-line medication initiated, n (%)^d^	20 (5.0)	20 (6.0)

^a^*P*=.001.

^b^*P*<.001.

^c^Includes notriptyline, amitriptyline, gabapentin, pregabalin, nabilone, dronabinol/sativex, and serotonin-norepinephrine reuptake inhibitors (SNRIs).

^d^Includes topical lidocaine, tramadol, opioids, methadone, selective serotonin reuptake inhibitors (SSRIs) and anticonvulsants not included as first line.

Finally, the use of first-line medication was higher than the use of second-line medication, even at baseline. There was no significant change in the initiation of either first-line or second-line medications from pre-CDSS to post-CDSS periods.

When analyzing by patient, rather than the encounters, we found that 56 of the 100 patients in the pre-CDSS sample and 69 of the 100 patients in the post-CDSS sample were either newly prescribed or already taking a first-line neuropathic pain medication. Although this suggests an increase in appropriate prescribing over the study year, the difference did not reach statistical significance, at *P*=.06.

### Utilization of the Clinical Decision Support System

At the 12-month time point, 18 of 169 possible clinicians (10.7%) had saved data in the CDSS neuropathic pain forms. A total of 1352 fields were saved on 40 neuropathic pain forms. Utilization was highest among nurse practitioners, with an average of 61 forms saved per nurse, 12 forms per resident, and 5.4 forms per physician.

Among study participants, both those who used any of the CDSS forms and those who did not, showed significant increases in awareness and adoption of guideline recommendations. Users and nonusers had similar awareness and adoption at baseline.

### Experience of Using Clinical Decision Support System

Among the 5 focus groups at 3 sites, there were 23 participants, including 10 physicians, 10 residents, and 3 nurse practitioners. Participants had all been introduced to the CDSS either through in-person or online training or through contact with colleague champions. Several key themes were evident.

Access and workflow were commented on most frequently. Owing to the combination of the newer format of CDSS and the older format of user interface, people had difficulty finding the CDSS link. In addition, its integration with other chronic disease management tools was an unfamiliar feature, and they did not completely trust its function. As one participant reported, “...I thought it was a great idea and I was enthusiastic...if it takes more than 3 seconds or something like that, it quickly falls off your priority list to do.”

The format and function of the CDSS itself was appealing once they had this open. However, some found that there were more information and data fields available than what they wished to make use of. Some felt that the number of patients they managed with pain was too low to develop ease with using the CDSS. A participant reported, “I remember going in there and clicking around and finding all the different things that were in there and I think if I had spent more time in there and used it, it probably would have been valuable.”

Most had opened and referred to the CDSS for the guideline recommendations 1 or more times, even if they had not entered data or used the dynamic functions. Reference information related to guideline recommendations was generally valuable and well accessed. Many participants had opened the CDSS to view the material there, often to confirm that their practice was fitting with guidelines. This was particularly common for first year residents and for nurse practitioners. As one resident said, “...when I have read it through enough times it gave me that practice and just, just asking those questions. So, I use it more as a reference tool I think.”

## Discussion

### Principal Findings

Our priority was to create a CDSS that was appealing to clinicians and therefore would be used in a way that would translate knowledge into practice. We also discovered that clinician knowledge concerning guideline recommendations for neuropathic pain was higher in our study participants at baseline than anticipated, leaving less room for improvement. In spite of this, there were several significant findings that will help to inform future CDSS development.

Utilization of the CDSS for data entry, and thus the dynamic decision support functions, was low, although the utilization for reference information was higher. In addition, utilization was much higher among nurses and first year family medicine trainees. All of this suggests that, for this type of clinical scenario at least, the value of a CDSS is highest for its reference material and for its influence on developing new practice patterns and behaviors. Once those patterns and behaviors are developed, the CDSS is less appealing, likely in part as its recommendations do not change.

Our study aimed to improve our understanding of the impact of a CDSS on patient outcomes. Improvements in knowledge translation were observed from several perspectives. Self-reports of awareness and adoption of recommendations showed statistically significant improvement. Behaviors observed through chart audit showed that the assessment of pain specifically for neuropathic pain had a statistically significant increase as well. These outcomes are valuable as they are indicators of the quality of care. Ultimately, we would also hope to see an increase in the appropriate use of medication. We discovered that a majority of these patients were using first-line medications already at baseline, and that although there was a shift to even higher use of first-line medication in the year of observation, this was not statistically significant. As the utilization of the data fields in the CDSS was low, it seems unlikely that we can attribute any improved practice to the data collection aspect of the CDSS. Some combination of the training activities and the passive reference material included in the CDSS are more likely to have influenced practice.

### Strengths and Limitations

We created a CDSS taking into account factors that have been shown to lead to the success of a CDSS, such as being created by study authors rather than by an external vendor, providing decision support at the time and location of decision making, and integration into practice workflow [2,13-15]. Our prototype included significant enhancements of the system interface that improved visibility and integration of the CDSS with usual work flow. This proved highly effective in usability testing [22]. However, production deployment of the modified interface was not complete by the time of this research project; therefore, optimal integration with the user interface was not achieved.

In addition, our CDSS is only minimally responsive to specific features of individual patients. Most features of this system would be typical of a recommendation system, rather than a decision support system. It may be most useful therefore as a training tool rather than for ongoing decision support. Neuropathic pain is a relatively rare entity in primary care, making it difficult to study [20]. Finally, our observational study design does not allow us to attribute any improved quality of care to the introduction of the CDSS itself.

### Conclusions

Our study demonstrated that aligning all necessary dimensions of information systems development to meet research timelines, while achieving measurable impact on quality of care, is challenging. We were able to demonstrate improvement in clinical practice that may have resulted from clinicians developing practice patterns learned from recommendations included in the CDSS. Ongoing use of the CDSS was not common.

## Abbreviations

CDSS: clinical decision support system
CPG: clinical practice guideline
EMR: electronic medical record
LBP: low back pain
OSCAR: Open Source Clinical Application Resource
VA: Veterans Affairs
